# Increased frequency of germline *BRCA2* mutations associates with prostate cancer metastasis in a racially diverse patient population

**DOI:** 10.1038/s41391-018-0114-1

**Published:** 2018-12-12

**Authors:** Gyorgy Petrovics, Douglas K. Price, Hong Lou, Yongmei Chen, Lisa Garland, Sara Bass, Kristine Jones, Indu Kohaar, Amina Ali, Lakshmi Ravindranath, Denise Young, Jennifer Cullen, Tiffany H. Dorsey, Isabell A. Sesterhenn, Stephen A. Brassell, Inger L. Rosner, Doug Ross, William Dahut, Stefan Ambs, William Douglas Figg, Shiv Srivastava, Michael Dean

**Affiliations:** 1 Department of Surgery, Center for Prostate Disease Research, Uniformed Services University of the Health Sciences, Rockville, MD USA; 2 John P Murtha Cancer Center, Walter Reed National Military Medical Center, Bethesda, MD USA; 3 Genitourinary Malignancies Branch, National Cancer Institute, Bethesda, MD USA; 4 Division of Cancer Epidemiology and Genetics, National Cancer Institute, Bethesda, MD USA; 5 Urology Service, Walter Reed National Military Medical Center, Bethesda, MD USA; 6 Laboratory of Human Carcinogenesis, National Cancer Institute, Bethesda, MD USA; 7 Joint Pathology Center, Silver Spring, MD USA; 8 Present Address: St. Luke’s Boise Medical Center, Mountain States Urology, Boise, ID USA

**Keywords:** Cancer screening, Cancer genetics

## Abstract

**Background:**

Germline mutations in *BRCA2* have been linked to a higher risk of prostate cancer (PCa), and high frequency of *BRCA1* and *BRCA2* (*BRCA1/2*) gene alterations was recently reported in metastatic castration-resistant PCa specimens. Mutations in *BRCA2* vary in racial and ethnic groups including African-American (AA) and Caucasian-American (CA) populations.

**Methods:**

*BRCA1* and *BRCA2* genes were sequenced (Ion AmpliSeq targeted sequencing) in archived blood DNA specimens in 1240 PCa patients, including 30% AA patients, in three different cohorts: localized early stage (T2) PCa (*N* = 935); advanced PCa (50% T3–4) (*N* = 189); and metastatic PCa (*N* = 116). The sequences were analyzed for known and novel mutations in *BRCA1/2*. Statistical analyses were performed to determine associations of the mutations with clinico-pathological parameters.

**Results:**

*BRCA2* mutations with known pathogenic annotation were significantly more prevalent in men with advanced and metastatic PCa (3.1%) compared to patients with an organ-confined disease (0.7%). AA patients carried more frequently *BRCA1/2* variants of unknown significance (VUS) when compared to Caucasian Americans (4.6 vs. 1.6%, respectively). Significantly, pathogenic BRCA2 mutations in men with localized early stage PCa increased the risk of distant metastasis.

**Conclusions:**

Germline variants of unknown significance in *BRCA1/2* are more frequent in AA than CA PCa patients; however, the prevalence of pathogenic mutations were similar across the races. Patients carrying *BRCA2* pathogenic mutations are more likely to progress to metastasis.

## Introduction

DNA damage repair genes (DDRGs) play a critical role in protecting genome integrity whereas the presence of germline mutations predispose to several cancer types (e.g., breast, ovarian). In prostate cancer (PCa), several recent studies described DDRG mutations that associated with aggressive disease. Exome and targeted sequencing-based findings for metastatic castration-resistant prostate cancer (mCRPC) revealed the importance of mutations and copy number changes in DDRG genes (including *BRCA2, ATM, BRCA1, CHEK2, FANCA, RAD51B/D, PALB2*, and *MSH2*) for disease progression [1–3]. Moreover, the IMPACT study demonstrated that germline *BRCA1/2* mutations can be used to identify men with higher risk of developing PCa, and are associated with a more aggressive phenotype and poorer outcome [4, 5]. Thus, there is accumulating evidence that *BRCA1/2* mutation carriers are at an increased risk for aggressive PCa. Because poly (ADP-ribose) polymerase (PARP) inhibitors can extend overall survival in mCRPC patients with DDRG mutations [6], the targeted identification of *BRCA1/2* mutation carriers could improve survival in this group of patients.

Variants in *BRCA1*/*2* are categorized into the following groups: pathogenic alleles that either inactivate the protein or are documented to confer risk for cancer, variants of unknown significance (VUS) that are as yet unclassified, likely benign and benign variants that are not known to confer cancer risk. However, this classification has been largely established for breast cancer patients and families; whether the same functional relationships exist for PCa is unclear. More studies are needed to establish if and how mutation frequencies in *BRCA1* and *BRCA2* vary in racial and ethnic groups including African-American (AA) and Caucasian-American (CA) populations [7]. It is important to note that AA men have the highest mortality rate from PCa [8] and their tumor biology remains understudied. Therefore, we included AA PCa patients into this study who were recruited either within the Department of Defense (DOD) Health Care System [9] or the National Cancer Institute (NCI) Maryland Case-Control Study [10], allowing for an evaluation of *BRCA1/2* mutation frequencies in AA and CA men with PCa. An understanding for the role of racial/ethnic differences of *BRCA1/2* variants in PCa is important and is also urgently needed because there is a persistent underrepresentation of samples from minorities in public databases (ClinVar, ENIGMA).

## Materials and methods

### Patient recruitment and characteristics

Germline DNA was obtained from 935 men (cohort 1) who were recruited at the Walter Reed National Medical Military Center (WRNMMC) (Supplementary Table 1). These patients were treated with radical prostatectomy between 1996 and 2012 for very low-, low-, and intermediate-risk PCa, according to National Comprehensive Cancer Network (NCCN) guidelines and were enrolled in the Center for Prostate Cancer Research (CPDR) longitudinal database and this study, under an Institutional Review Board (IRB)-approved protocol. Additional DNA samples were obtained from 189 participants in the NCI Maryland Prostate Cancer Case-Control study (cohort 2). This study recruited 976 AA and CA PCa patients between 2005 and 2016, as previously described [10]. Blood DNA samples were also obtained from participants in clinical trials for metastatic PCa at the National Cancer Institute (cohort 3, *N* = 116) (Supplementary Table 1). All studies were approved by IRBs of the individual institutions and written informed consent was obtained from all subjects.

### Ion AmpliSeq targeted gene sequencing

*BRCA1*/*2* genes were sequenced in archival blood DNA specimens (*N* = 1240) collected from PCa patients undergoing radical prostatectomy treatment at WRNMMC, from CPDR and from the NCI. Genomic DNA was extracted from peripheral blood lymphocytes using DNeasy Blood and Tissue DNA isolation kit. The DNA samples from the three studies were amplified and libraries prepared following the Ion Ampliseq^TM^ Library Preparation protocol. Individual samples were barcoded, pooled, applied to chips, and sequenced on the Ion Torrent^TM^ PGM Sequencer using the Ion PGM^TM^ Template OT2 200 and Ion PGM^TM^ Sequencing 200v2 kits. Mean read length after sequencing was 116 bp, and 94% of amplicons gave an average coverage of greater than 50 reads per sample. Resulting sequence reads were aligned to the human reference genome version hg19 using the TMAP aligner and single-nucleotide variants were called using both Genome Analysis Tool Kit (GATK) [11, 12] and Torrent Variant Caller (TSVC) as previously described [13]. The small insertions and deletions were called using the TSVC. All mutations, in Supplementary Table 1, were manually examined in Integrative Genomics Viewer (IGV) [14] to confirm an adequate number of mutant reads in both directions, and to eliminate false positives. The primer panel was previously validated against known mutations and in other studies [13]. We have previously compared the sensitivity of Ion Torrent sequencing and variant calling against Illumina and Complete genomics platforms and found them to be comparable [15]. The primer panel used here was previously shown to have over 99% coverage of the coding regions and splice sites of BRCA1 and 98% of BRCA2 [13]. Known mutations were used as positive controls and predicted mutations detected with this panel have been verified by Sanger sequencing in this and other studies [13]. The primer panel has been validated against a panel of 115 known variants and shown to have high sensitivity and specificity [16]. The sequences were analyzed for known and novel mutations in *BRCA1/2*, and variants less than 3% in frequency in the 1000 genomes database were selected for further analysis. Known variants were annotated with data from ClinVar to determine clinical relevance.

### Statistical analyses

The chi-square test was used to compare the distribution of the clinico-pathological characteristics between CA and AA patients, as well as by *BRCA1/2* mutation status. Unadjusted Kaplan–Meier estimate curves were used to evaluate prognostic significance. For all other data analysis, Student's *t*-test was used. *P* < 0.05 was considered to be statistically significant. All analyses were conducted using SAS software, version 9.3.

## Results and discussion

To examine the frequency of *BRCA1/2* germline variants and mutations in our cohort, we sequenced the two genes in early stage (T2) and advanced stage (T3–4 and metastatic) PCa patients and evaluated the impact of pathogenic *BRCA1/2* mutations on PCa progression and distant metastasis in subjects unselected for family history, in both AA and CA patients.

To identify and determine the frequency of *BRCA1*/*2* mutations in PCa, a total of 1240 germline DNA patient samples were amplified and sequenced (Supplementary Table 1). Variants in the *BRCA1/2* genes were categorized as benign/likely benign (together with wild type), VUS, and pathogenic. Non-coding variants are shown in Supplementary Table 4. The population was comprised of mainly CA subjects 69.2% (855/1235) but enriched for AA patients (28.6% (353/1235)), while 2.6% (32/1235) were patients from different race/ethnic background (Asian, Hispanic) (Supplementary Table 1). Being of African descent was self-reported, providing an opportunity to compare the *BRCA1/2* variant and mutational status between AA and CA patients. This comparison showed that AA patients had an increased frequency of germline VUS mutations in *BRCA1* and *BRCA2* genes compared to CA patients (4.6 vs. 1.6%, respectively); however, the prevalence of pathogenic mutations was similar (1.4 vs. 1.0%, respectively) (Table 1).

**Table 1:** Association of *BRCA1/2* germline mutations with race and tumor characteristics (combined three cohorts, *N* = 1240)

Variable	WT/benign/likely benign^a^	VUS^b^	Pathogenic^c^	*P* value
Race				
AA	412 (94.1)	20 (4.6)	6 (1.4)	0.0063
CA	750 (97.4)	12 (1.6)	8 (1.0)	
Clinical stage				
Early stage (T2)	984 (96.5)	29 (2.8)	7 (0.7)	0.0206
Advanced stage (T3–4/N1/M1/D3)	182 (94.3)	5 (2.6)	6 (3.1)	
Biopsy Gleason sum				
6 or less	659 (96.6)	19 (2.8)	4 (0.6)	0.0331
7	317 (95.5)	11 (3.3)	4 (1.2)	
8–10	169 (95.5)	2 (1.1)	6 (3.4)	

Numbers in parenthesis indicate % values

^a^Wild type *BRCA1/2* and benign/likely benign mutations in *BRCA1/2*

^b^Variants of unknown significance in *BRCA1/2*

^c^Pathogenic mutations in *BRCA1/2*

Next, we analyzed the relationship between *BRCA1/2* mutations and disease status. Our results show that there is a wide spectrum of germline mutations in *BRCA1/2* and confirm that only a small percentage of men treated for localized PCa are carriers for *BRCA1*/*2* mutations (7 of 1020 subjects carried pathogenic variants, 0.7%) [17]. However, as previously indicated, in patients with metastatic and advanced PCa, this percentage is significantly increased (over 4-fold) to 3.1% (6 of 193) (Table 1). In advanced PCa patients most of the pathogenic mutations were found in the *BRCA2* gene (85.7%, 6 of 7 in *BRCA2* vs. 14.3%, 1 of 7 in *BRCA1*). The Ashkenazi *BRCA1* c.68_69delAG (185delAG) and *BRCA2* c.5946delT (6174delT) founder mutations were each found in one metastatic PCa patient (Supplementary Table 2). All 19 pathogenic mutations in *BRCA1* and *BRCA2* are depicted in a schematic map format (Supplementary Fig. 1). An even higher frequency (6.0%, 9/150) of *BRCA1/2* germline mutations was reported in a large multi-center study of CRPC [3]. Our data add to the increasing evidence that *BRCA1/2* mutation carriers develop more aggressive PCa [18, 19].

The largest subset of the PCa patients (CPDR cohort, *N* = 935) in this study was treated by radical prostatectomy for localized PCa in an equal access DoD health care system (CPDR/WRNMMC). Mutations in *BRCA1/2* were detected in 19% of PCa patients; however, most of them (over 15%) were categorized as benign or likely benign. Pathogenic or VUS mutations affected 35 of the 935 evaluable PCa patients (3.7%) (Supplementary Table 1). No significant association of *BRCA1/2* mutations with ERG (erythroblast transformation-specific (ETS)-related gene) status of the tumor was apparent, although the number of pathogenic mutations was too low to conclude. Co-morbidity (other cancer) was not associated with *BRCA1/2* mutations (Data not shown).

**Fig. 1:**
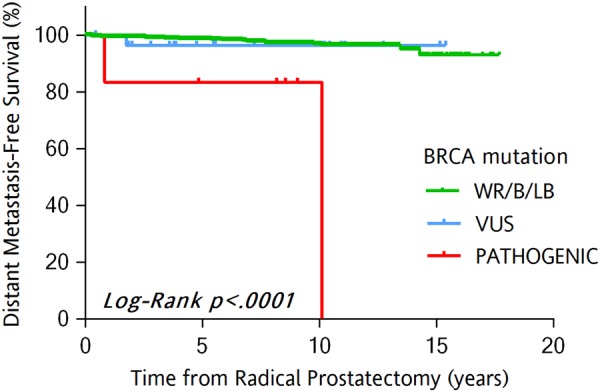
Kaplan–Meier distant metastasis-free survival curves across *BRCA1/2* mutation status (cohort 1, *N* = 927). The figure depicts time to distant metastasis as survival estimate for the categories of patients based on *BRCA1/2* mutation status. **a** Wild-type *BRCA1/2* and benign/likely benign mutations in *BRCA1/2;* **b** variants of unknown significance in *BRCA1/2;* **c** pathogenic mutations in *BRCA1/2;* **d** metastasis

Most important, in this cohort with up to 20 years of follow-up time, pathogenic *BRCA1/2* mutations associated with future distant metastasis and shorter time to metastasis (Kaplan–Meier analysis, *p* < 0.0001, Fig. 1), but had no significant association with biochemical recurrence (Supplementary Fig. 2). At the time of radical prostatectomy patients with higher pathologic T stage (pT3–4 vs pT2) had an increased percentage of pathogenic mutations, but this increase was not significant (Supplementary Table 3). A limitation of this study is that we did not use a method such as MLPA (multiplex ligation-dependent probe amplification) [20] to identify large insertions and deletions.

In summary, patients carrying pathogenic *BRCA* mutations, most frequently in *BRCA2*, confer a higher risk of more aggressive PCa phenotype, and a higher risk of metastasis with a shorter survival time. Increased frequency of VUS mutations in *BRCA* genes of AA patients, compared to CA patients, suggest that it may be important to evaluate these VUS mutations for potential pathogenic effects in PCa. Recent studies demonstrate that PARP inhibitors (inhibiting single-strand DNA break repair), such as Olaparib, can slow progression-free survival and extend overall survival in patients with *BRCA1/2* mutations [21]. Therefore, testing for *BRCA1/2* germline mutations in high-risk patients (familial history or tumor with poor prognostic clinico-pathologic features) may provide useful information for treatment stratification and for improving current nomograms.

## Disclaimer

The views expressed in this article are those of the author and do not reflect the official policy of the Department of Army/Navy/Air Force, Department of Defense, or the U.S. Government. The identification of specific products, scientific instrumentation, or organization is considered an integral part of the scientific endeavor and does not constitute endorsement or implied endorsement on the part of the author, DoD, or any component agency.

## Electronic supplementary material

Supplementary Table 4

Supplemental tables

sup fig 1

sup fig 2
