# Supplementary material for: Increased frequency of germline BRCA2 mutations associates with prostate cancer metastasis in a racially diverse patient population
Source: Prostate Cancer Prostatic Dis. 2018 Dec 12;22(3):406–10. doi: 10.1038/s41391-018-0114-1 (PMC6760554; doi:10.1038/s41391-018-0114-1)
Supplement: Supplementary file 2 — Supplemental tables [file 41391_2018_114_MOESM2_ESM.zip]

**Supplementary Table S1. Descriptive statistics of patients with evaluable *BRCA1/2* data in the three cohorts**

|  | **Cohort 1** | **Cohort 2** | **Cohort 3** |
| --- | --- | --- | --- |
| N | 935 | 189 | 116 |
| Age at diagnosis (year) |  |  |  |
| Mean (SD) | 58.5 (7.9) | 62.9 (8.6) | 58.6 (7.7) |
| PSA (ng/mL) |  |  |  |
| Median (range) | 4.9 (0.2-129.1) | 8.5 (1.4-1801.5) |  |
| Race |  |  |  |
| CA | 645 (69.0) | 137 (72.5) | 73 (62.9) |
| AA | 263 (28.1) | 52 (27.5) | 38 (32.8) |
| Other | 27 (2.9) |  | 5 (4.3) |
| Stage |  |  |  |
| Early stage (T2) | 897 (98.6) | 99 (52.4) | 24 (21.0) |
| Advanced stage (T3-4/N1/M1/D3) | 13 (1.4) | 90 (47.6) | 90 (79.0) |
| Missing | 25 |  | 2 |
| Biopsy Gleason |  |  |  |
| 6 or less | 620 (69.6) | 47 (24.9) | 15 (13.5) |
| 7 | 216 (24.2) | 86 (45.5) | 30 (27.0) |
| 8-10 | 55 (6.2) | 56 (29.6) | 66 (59.5) |
| Missing | 44 |  | 5 |
| Genotype |  |  |  |
| WT / B / LB* | 900 (96.3) | 183 (96.8) | 108 (93.1) |
| VUS | 29 (3.1) | 2 (1.1) | 4 (3.4) |
| Pathogenic** | 6 (0.6) | 4 (2.1) | 4 (3.4) |

Numbers in parenthesis indicate % values.

* Wild type / Benign / Likely Benign

** Cohort 1: 4 out of 6 pathogenic mutations in *BRCA2*, 2 in *BRCA1*

** Cohort 2: 3 out of 4 pathogenic mutations in *BRCA2,* 1 in *BRCA1*

** Cohort 3: 3 out of 4 pathogenic mutations in *BRCA2,* 1 in *BRCA1*
