# Supplementary material for: Increased frequency of germline BRCA2 mutations associates with prostate cancer metastasis in a racially diverse patient population
Source: Prostate Cancer Prostatic Dis. 2018 Dec 12;22(3):406–10. doi: 10.1038/s41391-018-0114-1 (PMC6760554; doi:10.1038/s41391-018-0114-1)
Supplement: Supplementary file 3 — sup fig 1 [file 41391_2018_114_MOESM3_ESM.zip]

**Supplementary Figure S1. Pathogenic mutations identified in *BRCA1* and *BRCA2***

Seven pathogenic mutations in *BRCA1* and twelve pathogenic mutations in *BRCA2* are shown in a schematic map format. The type of mutations are indicated under the map.

**
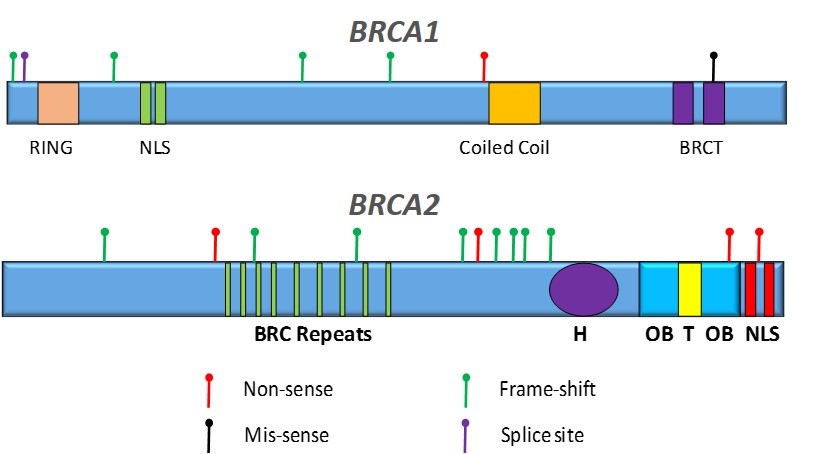
**
