# Supplementary material for: Increased frequency of germline BRCA2 mutations associates with prostate cancer metastasis in a racially diverse patient population
Source: Prostate Cancer Prostatic Dis. 2018 Dec 12;22(3):406–10. doi: 10.1038/s41391-018-0114-1 (PMC6760554; doi:10.1038/s41391-018-0114-1)
Supplement: Supplementary file 4 — sup fig 2 [file 41391_2018_114_MOESM4_ESM.docx]

**Supplementary Figure S2. Kaplan-Meier BCR-free survival curves across *BRCA1/2* mutation status (Cohort 1, N=908)**


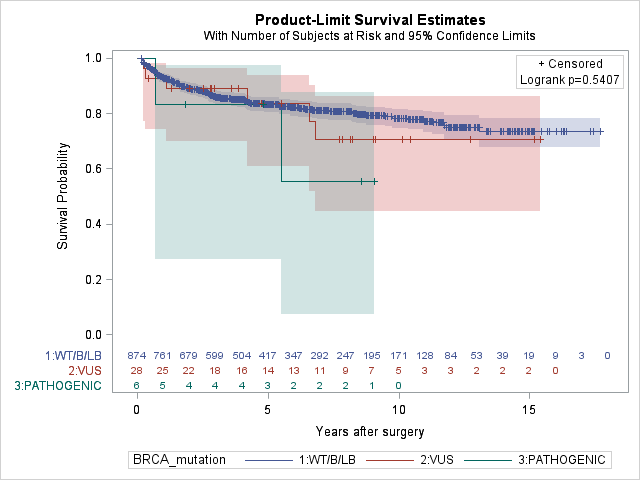


| BRCA mutation status | Non-BCR | BCR |
| --- | --- | --- |
| WT/Benign/likely benign | 722 (82.6) | 152 (17.4) |
| VUS | 22 (78.6) | 6 (21.4) |
| Pathogenic | 4 (66.7) | 2 (33.3) |

Numbers in parenthesis indicate % values.
